# Supplementary material for: Comparative Antennal Morphometry and Sensilla Organization in the Reproductive and Non-Reproductive Castes of the Formosan Subterranean Termite
Source: Insects. 2021 Jun 24;12(7):576. doi: 10.3390/insects12070576 (PMC8307099; doi:10.3390/insects12070576)
Supplement: Supplementary file 1 [file insects-12-00576-s001.zip › Supplementary materials/Supplementary infomation_Figure S1.pdf]

Supplementary Information

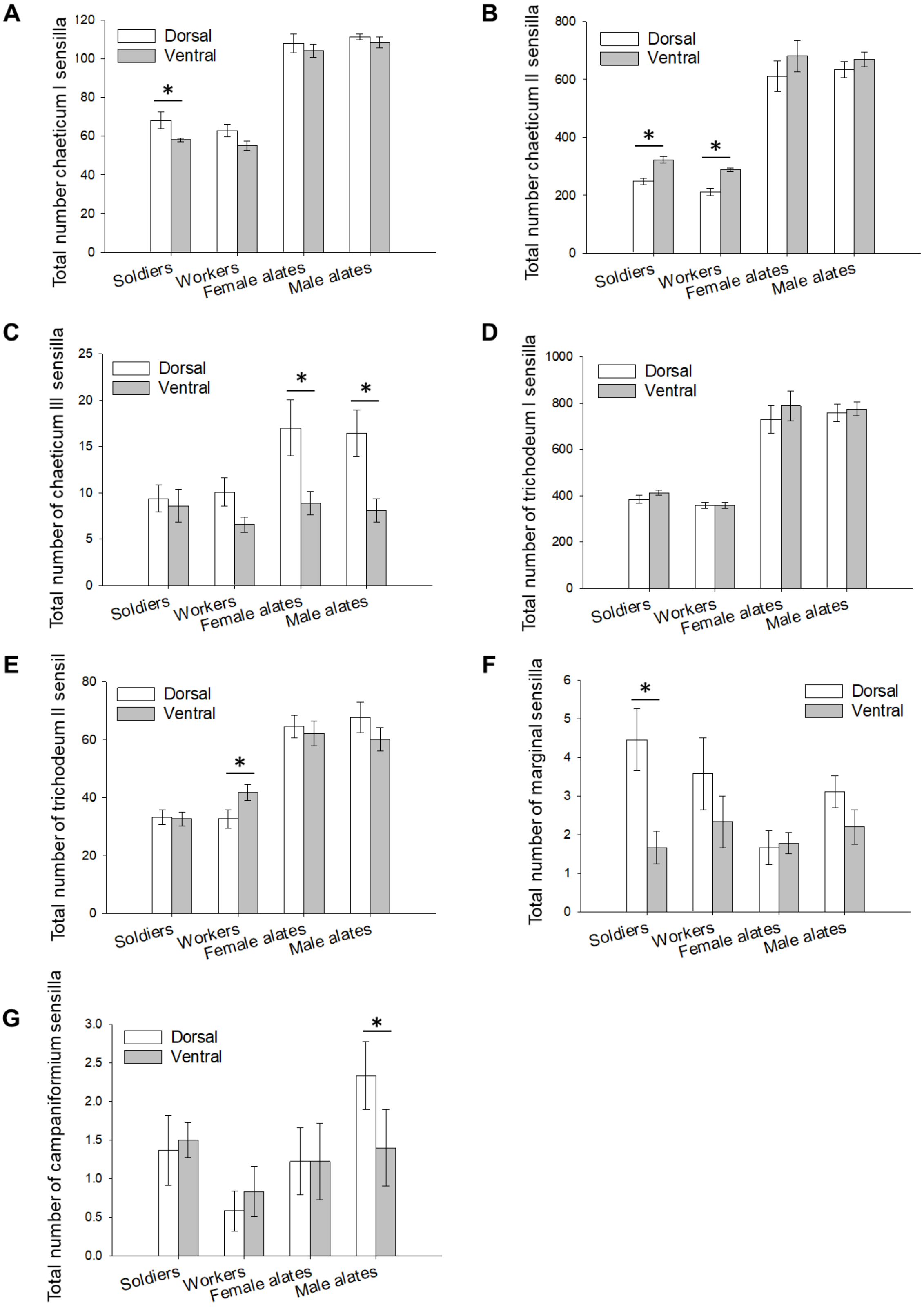

Figure S1. Dorsal and ventral distribution of sensilla chaeticum I (A), chaeticum II (B), chaeticum III (C), trichodeum I (D), trichodeum II (E) marginal (F), and campaniformium (G). Total number of sensilla on dorsal and ventral sides per antenna are shown (mean ± SE; *, $P < 0.05$; unlabeled pairs, $P > 0.05$; Wilcoxon rank sum tests; dorsal side: n = 11, 12, 9, and 9 for soldiers, workers, female and male alates, respectively; ventral side: n = 12, 12, 9, and 9 for soldiers, workers, female and male alates, respectively).
